# Built environmental characteristics, physical activity, diet, and nutritional status in Accra, Ghana: a cross-sectional study

**DOI:** 10.1186/s12889-026-26511-4

**Published:** 2026-01-31

**Authors:** Emmanuel Sintim Effah, Abraham Nai, Helga Bárdos

**Affiliations:** 1 Doctoral School of Health Sciences, University of Debrecen, Debrecen, Hungary; 2 Department of Public Health and Epidemiology, Faculty of Medicine, University of Debrecen, Debrecen, Hungary

**Keywords:** Built environment, Physical activity, Lower middle-income countries

## Abstract

**Background:**

The high prevalence of noncommunicable diseases related to lifestyle, diet, and obesity is a global health concern, including in lower-middle income countries. There is increasing evidence that characteristics of the built environment can influence residents’ lifestyle, including physical activity and diet. The aim of this study is to describe the characteristics of the built environment and the lifestyle and nutritional status of residents living in different neighborhoods of Accra, Ghana.

**Methods:**

A cross-sectional study was conducted in Accra between June and July 2023. Twelve neighborhoods were randomly selected on the basis of high and low values of residential density and socioeconomic status. Sociodemographic, household and neighborhood information was collected using the Neighborhood Environment Walkability Scale (NEWS-A) questionnaire adapted for Africa. Physical activity was assessed by the WHO Global Physical Activity Questionnaire. Differences between variables by neighborhood types were analyzed using Chi square test and Kruskal Wallis test, and pairwise comparisons were performed by Dunn-Bonferroni test.

**Results:**

A total of 631 adults (median age 34.0 years, female 60.5%) participated in the survey. The built environmental characteristics of the different neighborhood types showed significant differences in all subscales of the NEWS (land use mix, street connectivity, safety for walking and cycling, aesthetics, safety from traffic, safety from crime, personal safety, and stranger danger). Work, transport-related and leisure-time physical activity levels and diet (consumption of fruits and vegetables, sweets, sugar-sweetened beverages, fast food and street food, alcohol) revealed variations between neighborhoods. Differences in nutritional status were inconsistent across neighborhood types.

**Conclusions:**

This study was the first to examine the associations between environmental characteristics, physical activity, diet and BMI in an urban setting in Ghana. Initial descriptive results revealed significant differences in health behaviors and the characteristics of the built environment between neighborhoods in Accra. The associations between these factors require further analysis.

## Background

The increasing prevalence of noncommunicable diseases related to lifestyle, diet, and obesity has become a global public health concern in recent decades [1]. According to the WHO report, 43% of adults worldwide were overweight, and 16% were obese in 2022. Global adult obesity prevalence has more than doubled between 1990 and 2022 [2]. In developing countries the narrative of the double burden of malnutrition, i.e., overweight/obesity and underweight, has been steadily changing in recent years, with underweight levels decreasing and overweight and obesity levels significantly increasing [3] However, overweight and obesity are increasing more rapidly in lower-middle-income countries, close to the levels observed in higher income countries [4, 5]. According to the Ghana Demographic and Health Survey (DHS), between 1993 and 2014, obesity among women (aged 15–49 years) increased from 3.4% to 15.3% over 21 years [6]. The latest Ghana DHS 2022 survey revealed that half of the women (50%) are either overweight or obese (28% are overweight and 22% obese), whereas 5% are underweight. Among men between the ages of 20 and 49, the prevalence of underweight is 5%, whereas 21% are either overweight or obese (17% are overweight and 4% are obese) [7].

These alarming trends in the obesity epidemic highlight the need to examine contextual factors, such as the built environment, that influence obesity-related behaviors [8, 9]. Systematic reviews showed two factors of the physical environment consistently linked to weight status, which are urban sprawl and land-use mix. Sprawling, low density areas tend to promote obesity by discouraging active travel and limiting access to amenities. Diverse land use generally supports lower obesity rate by providing greater opportunities for active travel [10–13]. High residential area density areas are typically well connected, with nearby destinations and supportive infrastructure for walking, cycling, and public transport [10]. The food environment is also recognized as a key factor in the prevention of obesity. Obesity levels were higher in areas with predominantly unhealthy or ultra-processed food outlets, whereas greater availability of fruits, vegetables, and supermarkets—facilitating healthier diets—was linked to lower levels of obesity [14].

In developed economies, obesity-related behaviors and prevalence exhibit a social gradient, with higher rates in socioeconomically disadvantaged neighborhoods [15]. In contrast, in low-income countries, obesity is more common among the affluent and educated people, whereas in middle-income countries the association is mixed for men and predominantly negative for women [16]. 

Studies have shown that certain built environment constructs, such as residential density, crime, traffic safety, availability of physical activity facilities, and infrastructure, play crucial roles in influencing physical activity levels. Improving the characteristics of built environments such as footpaths (provision and quality), safety (from traffic and crime) and aesthetics (clean, litter- and graffiti-free streets, presence of plants and trees) can potentially increase transport-related physical activity and has been considered a low-cost and effective way to improve health [17, 18].

The impact of the built environment on physical activity, diet, obesity, and health in Africa has been less studied. Perceived neighborhood safety from crime and traffic was observed to be related to physical activity among adults in Nigeria [19]. Others have reported no associations between GIS-measured walkability and physical activity in South Africa [20]. A recent systematic review on the associations between built environment constructs and physical activity among children and adolescents in Africa, which was based on 6 studies, revealed associations between traffic and crime safety and physical activity [21].

Only a few studies have investigated the neighborhood environment in Ghana. Housing and living conditions were reported to be correlated with the health status of the occupants in Accra [22]. The results of the Ghana Obesity Survey 2021 revealed sociodemographic determinants of physical activity, diet, and obesity among adults in Ghana but did not measure the built environment characteristics of neighborhoods [23]. However, it has been reported that the local food environment, which is based on GPS positioning, is associated with an increased risk of obesity through access to convenience stores in urban poor Accra [24].

To our knowledge, no studies have been conducted in Ghana to measure the perceived built environment characteristics of urban neighborhoods and physical activity, diet and BMI among adults. Therefore, the aim of this study was to describe the characteristics of the built environment and physical activity, diet and nutritional status of residents in different neighborhoods of Accra, Ghana.

## Methods

### Study design

A cross-sectional study was conducted within the Accra Metropolitan Assembly between June and July 2023.

### Study area

The study was conducted within the Accra Metropolitan Assembly in the Greater Accra region. More than 1.6 million people live in the Accra Metropolitan Assembly, with a daily influx of 2 million business commuters. Accra is in the Greater Accra region, the capital of Ghana. Greater Accra region is considered to be the most urbanized, diversed and the most densely populated in Ghana with the proportion of population that is urban to be 91.7% with household size of over 1.57 million [25–27].

### Sampling design

The sampling of neighborhoods was based on a combination of residential area density and socioeconomic status at the neighborhood level. The neighborhood residential area density was calculated considering the number of populations per square km [28]. The socioeconomic status of neighborhoods was based on the slum index, which captures aspects such as inadequate housing, lack of essential services, and insecure tenure. These criteria are aligned with the UN-Habitat definition of slums [29].

GIS data (shape files) on the slum index and residential area density of Accra’s neighborhoods were obtained through the kind generosity of Professor J. Weeks [30, 31]whose research team used census survey data from the Ghana Statistical Service [32]. Attribute tables containing data on the slum index and residential density of neighborhoods were exported into Excel tables via the use of QGIS software [33]. Data were categorized into tertiles, and the upper and lower tertiles were selected to represent low and high socioeconomic status (SES) and low and high residential area density (RAD), respectively. Neighborhoods were stratified into four strata: high-SES/high-RAD, high-SES/low-RAD, low-SES/high-RAD, and low-SES/low-RAD. Three neighborhoods were randomly selected from each stratum, representing the 4 types of neighborhoods. Fifty households were systematically selected from the 12 neighborhoods included in the sample, with 1 in every five households. One adult from each household volunteered to participate in the study.

**Fig. 1:**
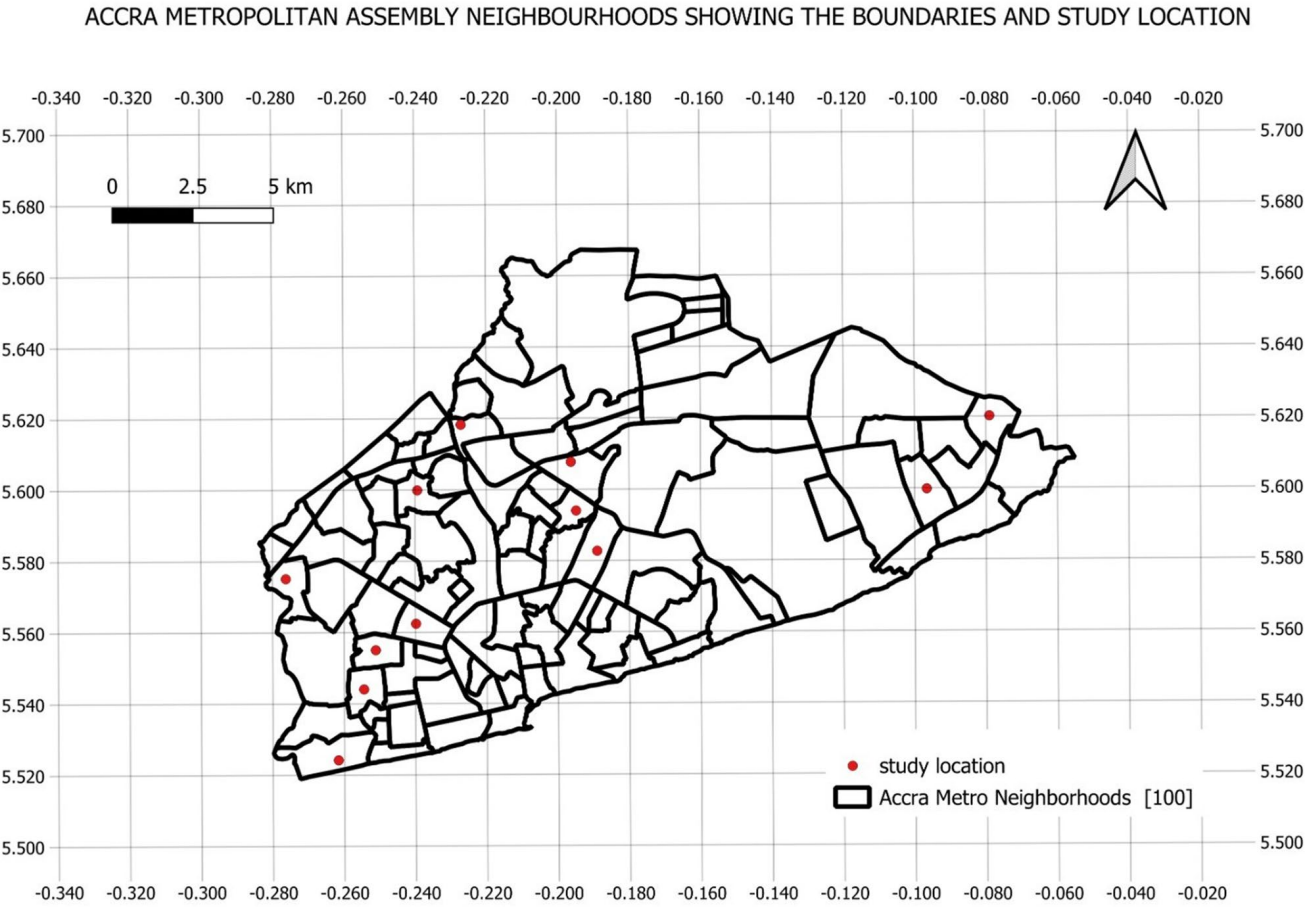
Map showing neighborhood boundaries of Accra Metropolitan Assembly, with study locations labeled in red dots

Figure 1 shows neighborhoods of Accra Metropolitan Assembly and study sites randomly selected.

### Sample size

The required sample size was calculated by using simplified formula for proportion the sample size [34]

$$\:n=\frac{N}{1+N({e)}^{2}}$$

The letter ‘n’ in the formula denotes the sample size ‘N’ the population size (> 100,000), ‘e’ denotes level of precision (0.05) for a 95% confidence interval, ‘+’ is the addition sign, and ‘^2^’ is the square sign.

$$\:n=\frac{100000}{1+100000\:({0.05)}^{2}}$$

*n* = 398 However, in the event of nonresponse, registration error, and to improve precision in subgroup analysis, ensure adequate representation of smaller subgroups and maintain at least 80% statistical power, based on the available logistics we increased the desired sample size to 600.

### Data collection

Information on the individual characteristics of the participants was collected through an interview-based survey conducted by researchers (ESE, AN) and two trained research assistants. Questions were asked to participants in English or interpreted in the local language, and responses were recorded on tablets using KOBO software [35]. All participants provided informed consent, which was recorded electronically. The researcher read and explained the consent form to participants who could not read or write in the local language, confirmed that they understood, and obtained their consent. A literate witness was present when the researcher explained the consent form to the respondent or when it was read aloud and explained to the respondents. Participants or literate witnesses could click on the ‘I agree’ box to indicate their consent to participate. Participants who could read and write gave their informed consent after reading and voluntarily agreeing to participate. During the survey, the COVID-19 protocols were followed in accordance with the regulations in force at the time.

### Perceived built environment

Perceived built environmental characteristics related to physical activity were assessed by the Neighborhood Environment Walkability Scale validated for Africa (NEWS-Africa) [36, 37] The NEWS-Africa is a validated adaptation of the original NEWS instrument [38]. It was developed to measure perceptions of neighborhood environmental features that influence physical activity in African contexts. Participants were recruited from Cameroon, Ghana, Kenya, Mozambique, Nigeria, South Africa and Uganda. It was developed through careful translation, cognitive testing and psychometric validation to ensure its relevance to both urban and rural environments across the continent. Over 95% of NEWS-Africa scores showed “excellent” (ICC > 0.75) or “good” (ICC = 0.60–0.74) reliability. In South Africa, NEWS-Africa was used in an urban study to examine the relationship between perceived walkability, physical activity and BMI among adolescents [39]. 

Our study deployed a total of 66 items from the original questionnaire, consisting of 10 computed subscales, to suit the research. These subscales include residential density (1 item), land use mix – destinations (20 items) – recreation (4 items), connectivity of streets (5 items), places for walking and cycling (12 items), aesthetics (8 items), safety from traffic (6 items), safety from crime (4 items), personal safety (3 items), and child-related inquiries on stranger danger (3 items). The details of the NEWS-Africa subscales are described below.

### Residential density

One item addressing residential density asked about the main type of housing in the immediate neighborhood of the respondents. The classifications and coding were (1) very few residential buildings within a 2–5 min walk from my house; (2) detached or semidetached family houses with space/garden; (3) attached (row) housing, apartment blocks/flats or multifamily housing with 2–5 stories; (4) multiple apartment blocks/flats of 6 stories or more with large spaces between buildings; (5) multiple apartment blocks/flats of 6 stories or more with very little space between buildings; and (6) densely populated small houses (1-story homes, including informal settlements and slums).

### Land use mix

The “land use mix–destination” scale was used to determine the perceived walking distance from home to 20 destinations (grocery, supermarket, fruit and vegetable vendor, fast food restaurant, street food vendor, pub or bar, cinema or theatre, place of worship, computer/cell phone kiosks, library, school, workplace or school, bookstore, healthcare clinic/hospital, pharmacy, hair dresser, clothing store, electronic shop, public bus stop, taxi/motor bike stop). “Land use mix – Recreation” asked about the perceived walking distance from home to recreational facilities (sports fields, other outdoor recreation spaces, indoor recreation facilities, and dance or martial arts classes). Proximity was measured on a 5-point scale, where a score of 1 represented proximity (less than a 5-minute walk), and a score of 5 represented considerable distance (more than a 30-minute walk). The average score of the responses was calculated. Scoring was reversed to be consistent with scoring on other scales. A score of 5 indicates the closest destinations and represents a higher land use mix compared to lower scores.

### Connectivity of streets

The agreement with five sentences related to distance to next street, alternative roads, unofficial roads, shortcuts, and blocked roads were rated on a 4-point scale. The average score was calculated, with higher scores of 4 indicate better street connectivity within the neighborhood, while lower scores of 1 indicate poor street connectivity.

### Places for walking and cycling

Agreement with 12 sentences related to the presence and quality of sidewalks, crosswalks, and bicycle lanes was rated on a 4-point scale. Average scores were calculated, with higher score of 4 indicating the presence of better facilities to support walking and cycling and lower scores 1 indicating poor facilities.

### Aesthetics

Aesthetics were the average of the rating on a 4-point agreement scale related to neighborhood surroundings (trees along the roads, clean, litter-free neighborhood, free from bad smells, free from unpleasant noises, free from pollution, presence of attractive buildings, beautiful natural sight and pleasant natural sounds). A higher average score of 4 indicated that the neighborhood surroundings were considered aesthetically pleasing and 1 indicated that the neighborhood’s aesthetics is very poor.

### Safety from traffic

Safety from traffic was rated on a 4 point scale. Statements were related to the volume and speed of traffic, exceeding the speed limit, and the dangers of walking, playing and cycling because of careless, aggressive, speedy driving. The average scores of the agreement scales were calculated, with higher score of 4 indicating a greater sense of safety from traffic and 1 indicates less safety from traffic.

### Safety from crime

Safety from crime were rated on a 4-point scale. Statements were related to the presence of crime in the neighborhood. (There is a lot of crime, too much crime to go out for walks and play during the day, or during the night, lots of gangs threaten). The coding was reversed. Higher scores 4 represent safety from crime and 1 represent less safety from crime.

### Personal safety

The personal safety scale consisted of the average rating of 3 items and were rated on 4-point scale. (I see and can talk to people when walking, no stray animals, well-lit neighborhood). Higher average scores 4 indicate greater perceived safety and 1 indicate lower perceived safety.

### Stranger danger

The respondents with children completed the stranger danger section to indicate their concern about their children’s safety during play and interactions. It consisted of 3 items and rated on 4-point scale (I am afraid of strangers around my house, I am afraid of strangers in my neighborhood, and I am afraid of strangers in a nearby park). Highest average score of 4 indicates a safest neighborhood for children and 1 indicates unsafe neighborhood for children.

### Social cohesion

We collected information on social cohesion with an adapted form of the Perceived Neighborhood Social Cohesion Questionnaire Brief Form (P-NSC-BF). It is a nine-item scale that captures three aspects of neighborhood social cohesion, namely, trust (“Most people in this area can be trusted.”), attachment (“People in this area have a high degree of community spirit”) and tolerance (“People in this area are tolerant of others who are not like them”). Agreement with the statements was measured using a 7-point Likert scale. Highest average scores 7 indicates highest social cohesion and 1 indicates lowest social cohesion [31].

### Physical activity

Physical activity was measured using modified WHO Global Physical Activity Questionnaire [40]. The content of the core questions is the same as the original GPAQ, but with the addition of an activity table showing examples of activities of different ranges and intensities. Providing these examples can help identify and include moderate to vigorous activities related to work, housework, transport, and leisure time. Work-related moderate and vigorous activity (work-related MVPA) consisted of 22 items and blank items for other nonspecific activities. Transport-related MVPA consists of two parts, walking and bicycling to and from places, with 8 items and blank items for all other unspecified activities. Leisure time-related MVPA included sports, fitness and other recreational activities, estimated separately for vigorous activities and moderate activities. The time spent with these activities per day was added to calculate work-related MPVA, transport-related MPWA, leisure-related MVPA and total MVPA in min/day.

### Diet

Questions were asked about the consumption of eight food items that are often used in food frequency questionnaires. Specifically, fruits, vegetables, fish, fast food, street food, sweets, and sugary soft drinks, including fruit juice and alcohol, are consumed. The answer options were as follows: once a week or less often, 2 times a week, 3 times a week, 4 times a week, 5 times a week, 6 times a week, 7 times a week, and two or more times a week. Based on the answers, we calculated the consumption frequency (times/week).

### Weight, height, BMI

The participant’s weight in kg was measured using a standard measuring scale, and their height was measured using a centimeter measuring rod. The participants were asked to remove their shoes and heavy clothing before weighing. BMI was calculated by dividing weight in kilograms by height in meters. Twenty participants refused to have their weight or height measured, so we could not calculate their BMI values.

### Sociodemographic variables

We asked questions about age, sex, marital status, educational level, employment status, income type, occupation, religion, ethnicity, number of children, and monthly income. Questions related to household ownership of motorized transport vehicles and the weekly use of private motorized transport (owned or owned by someone else) were also included. We also asked questions related to the number of adults in the household, the number of youths in the household, and the number of years spent in the household.

### Data analysis

We cleaned, coded, and analyzed the data with IBM SPSS 26.0. Descriptive characteristics and comparisons by neighborhood type were performed. The distribution of the data is presented as percentages for categorical variables and as medians (IQRs) or means (SDs) for continuous variables. Differences by neighborhood types were tested using the Chi-square test and the Kruskal‒Wallis test. Pairwise comparisons were performed using the Dunn-Bonferroni test. Significance levels were set at *p* < 0.05.

### Ethical approval and consent to participate

Approval for the research protocols and methodology was provided by the Ethical Review Committee of the Ghana Health Service. (GHS-ERC 022/03/23) The participants gave their written informed consent to each study population in accordance with the Declaration of Helsinki.

## Results

### Sociodemographic characteristics of the study participants

Table 1 describes the sociodemographic characteristics of the study participants by neighborhood types. The study sample consisted of 631 adults, and the proportion of females was slightly greater (60.5%) than that of males. The median age was 34.0 years, and more than half (52.8%) of the study participants were younger than 35 years. The proportion of females and the distribution of age groups showed no significant differences between the neighborhood types. Overall, 44.6% of the participants were married or lived with a partner, 45.1% were single, and 10.3% were divorced or widowed. A greater proportion of participants (55.1%) were married or lived with a partner in high-SES/high-RAD neighborhoods than in the other neighborhoods. The educational level of the participants did not significantly differ across neighborhoods. Approximately 11% of the participants reported no formal education, the same had primary education, two-thirds of the participants had secondary education, and 14% had tertiary education. Among the participants, self-employment (47.4%) was the most common, 26.4% were employed, and 26.2% were unemployed. Self-employment was most prevalent in high-SES/high-RAD neighborhoods, whereas employment rates were highest in high-SES/low-RAD neighborhoods, and unemployment rates were highest in low-SES/high-RAD neighborhoods. Approximately two-thirds of the participants worked in services and sales, as well as craft and related trade businesses. The primary source of income was informal (45.3%), followed by formal (30.2%). 24% of participants reported dependence (12%) or having no income (12%). Significantly more participants reported having income in high-SES neighborhoods than in low-SES neighborhoods. Most of the participants were Christians (81.1%) and belonged to the Akan and Ga-Dangme ethnic groups. 21% of participants’ households owned one or more vehicles. Approximately 24% of the participants used private (either their own or owned someone else) motorized transport (car, truck, motorcycle, or tricycle) on most days of the week.

**Table 1:** Sociodemographic characteristics of the study participants by neighborhood types

Variables	Categories	Total (*n* = 631)	High-SES/ High-RAD (*n* = 151)	High-SES/ Low-RAD (*n* = 152)	Low-SES/ High-RAD (*n* = 172)	Low-SES/ Low-RAD (*n* = 156)	*p*-value
Sex	Female	381 (60.5%)	84 (56.0%)	89 (58.6%)	117 (68.0%)	91 (58.3%	0.117
Age	18–35	330 (52.8%)	73 (50.0%)	80 (52.6%)	96 (56.1%)	81 (51.9%)	0.388
	36–50	166 (26.6%)	40 (27.4%)	40 (26.3%)	37 (21.6%)	49 (31.4%)	
	51–65	89 (14.2%)	27 (18.5%)	22 (14.5%)	23 (13.5%)	17 (10.9%)	
	65>	40 (6.4%)	6 (4.1%)	10 (6.6%)	15 (8.8%)	9 (5.8%)	
Marital status	Married or living with partner	281 (44.6%)	77 (51.3%)	67 (44.1%)	60 (34.9%)	77 (49.4%)	0.000
	Single	284 (45.1%)	62 (41.4%)	77 (50.6%)	79 (45.9%)	66 (42.3%)	
	Widowed or divorced	65 (10.3%)	11 (7.3%)	8 (5.3%)	33 (19.2%)	13 (8.3%)	
Education	No education	71 (11.3%)	18 (12.0%)	23 (15.1%)	16 (9.3%)	14 (9.0%)	0.163
	Primary	70 (11.1%)	19 (12.7%)	14 (9.2%)	18 (10.5%)	19 (12.2%)	
	Secondary	400 (63.5%)	93 (62.0%)	84 (55.3%)	118 (68.6%)	105 (67.3%)	
	Tertiary	89 (14.1%)	20 (13.3%)	31 (20.4%)	20 (11.6%)	18 (11.5%)	
Employment	Employed	166 (26.4%)	41 (27.3%)	53 (34.9%)	24 (14.0%)	48 (31.0%)	0.000
	Unemployed	165 (26.2%)	28 (18.7%)	44 (28.9%)	59 (34.3%)	34 (21.9%)	
	Self Employed	298 (47.4%)	81 (54.0%)	55 (36.2%)	89 (51.7%)	73 (47.1%)	
Income Type	Formal	190 (30.2%)	20 (13.3%)	74 (48.7%)	49 (28.3%)	47 (30.3%)	0.000
	Informal	285 (45.3%)	103 (68.7%)	44 (28.9%)	68 (39.5%)	70 (45.2%)	
	Dependent	77 (12.2%)	16 (10.7%)	14 (9.2%)	29 (16.9%)	18 (11.6%)	
	No income	77 (12.3%)	11 (7.3%)	20 (13.2%)	26 (15.3%)	20 (12.9%)	
Occupation	Services and sales	207 (32.8%)	59 (39.1%)	36 (23.7%)	48 (27.9%)	64 (41.0%)	0.019
	Craft and trade	186 (29.5%)	39 (25.8%)	58 (38.2%)	49 (28.5%)	40 (25.6%)	
	Professional	40 (6.3%)	7 (4.6%)	11 (7.2%)	11 (6.4%)	11 (7.1%)	
	Others	198 (31.4%)	46 (30.5%)	47 (30.9%)	64 (37.2%)	41 (26.3%)	
Religion	Christian	512 (81.1%)	126 (83.4%)	129 (84.9%)	126 (73.3%)	131 (84.0%)	0.089
	Muslim	105 (16.6%)	22 (14.6%)	19 (12.5%)	42 (24.4%)	22 (14.1%)	
	Others	14 (2.3%)	3 (2.0%)	4 (2.6%)	4 (2.3%)	3 (1.9%)	
Ethnicity	Akan	235 (37.3%)	63 (42.0%)	50 (32.9%)	43 (25.0%)	79 (50.6%)	0.000
	Ga-Dangme	140 (22.2%)	38 (25.3%)	25 (16.4%)	45 (26.2%)	32 (20.5%)	
	Ewe	123 (19.5%)	27 (18.0%)	45 (29.6%)	39 (22.7%)	12 (7.7%)	
	Mole- Dagbani	63 (10.0%)	11 (7.4%)	16 (10.5%)	28 (16.3%)	8 (5.1%)	
	Others	69 (11.0%)	11 (7.3%)	16 (10.6%)	17 (9.8%)	25 (16.1%)	
Vehicle(s) Owned	None	498 (78.9%)	107 (70.9%)	117 (77.0%)	157 (91.3%)	117 (75.0%)	0.000
	One or more	133 (21.1%)	44 (29.1%)	35 (23.0%)	15 (8.7%)	39 (25.0%)	
Vehicle use	Not at all or a few times	477 (76.2%)	107 (71.3%)	122 (80.3%)	118 (68.6%)	130 (85.5%)	0.001
	Most times	149 (23.8%)	43 (28.7%)	30 (19.7%)	54 (31.4%)	22 (14.5%)	

Differences in the distribution of sociodemographic characteristics across neighborhood types were statistically analyzed with a Chi square test reporting the number and percentages and *p* value

*SES* Socioeconomic status, *RAD* Residential area density

### Built environmental characteristics

Table 2 summarizes the built environment characteristics of neighborhoods measured using the Neighborhood Environment Walkability Scale for Africa (NEWS-Africa). Overall, the distribution of values at each NEWS scale showed significant differences between neighborhood types. The respondents from high-RAD neighborhoods reported more densely packed housing, especially in low-SES neighborhoods. Various destinations were generally close in proximity, within 10 minutes’ walk to households in neighborhoods, indicating a high land use mix. Proximity to recreational facilities was lower, being lowest in low-SES/high-RAD neighborhoods. The connectivity of streets was generally good but somewhat lower in high-SES/low-RAD neighborhoods characterized mainly by detached housing. Neighborhood aesthetics were usually rated as low but better in high-SES/low-RAD neighborhoods. The presence and quality of walking and cycling facilities were reported to be low but slightly better in high-SES/high-RAD neighborhoods. The respondents perceived overall low safety from traffic because of its high speed and volume, especially in low-SES/high-RAD neighborhoods. Safety from crime was generally perceived as low and was associated with the presence of gangs and crime, which were worst in low-SES/high-RAD neighborhoods. Personal safety was reported to be generally good because of the presence of people and lighting in the streets and the lack of stray animals. Overall, parents perceived danger from strangers to their children around the house in the neighborhoods, especially in low-SES/high-RAD neighborhoods.

**Table 2:** Built environmental characteristics by neighborhood types

NEWS SCALES	High-SES/High-RAD^a^	High-SES/Low-RAD^b^	Low-SES/High-RAD^c^	Low-SES/Low-RAD^d^	*p* value
Residential density (1–6)	3.00 (3.00–3.00)^b, c^	2.00 (2.00–3.00)^a, c, d^	6.00 (3.00–6.00)^a, b, d^	3.00 (2.00–3.00)^b, c^	0.000
Land use mix (1–5)					
Destination*	3.50 (3.08–3.76)^b^	3.13 (2.53–3.63)^a, d^	3.33 (2.94–3.67)	3.44 (3.08–3.74)^a^	0.000
Recreation*	3.00 (2.58-4.00)^c^	3.00 (2.00–4.00)^c^	2.00 (1.50-3.00)^a, b, d^	3.00 (2.33-4.00)^c^	0.000
Connectivity (1–4)	3.40 (3.40–3.64)^b, c, d^	3.10 (2.80–3.40)^a, c, d^	3.40 (3.20–3.40)^a, b^	3.40 (2.80–3.40)^a, b^	0.000
Aesthetics (1–4)	2.57 (1.86–3.04)^b, c^	3.00 (2.57–3.57)^a, c, d^	2.00 (1.14–2.92)^a, b, d^	2.71 (2.14-3.00)^b, c^	0.000
Safety (1–4)					
Walking and cycling	2.25 (1.92–2.83)^b, c, d^	2.00 (1.67–2.58)^a^	2.04 (1.54–2.58)^a^	2.17 (1.50–2.83)^a^	0.000
Traffic*	2.00 (1.67-3.00)^c^	2.33 (2.00-2.75)^c, d^	1.83 (1.33-2.00)^a, b, d^	2.00 (1.83–2.33)^b, c^	0.000
Crime*	2.00 (1.25-3.00)^b, c^	2.00 (2.00–3.00)^a, c, d^	1.75 (1.00–2.00)^a, b^	2.00 (1.00-2.75)^b^	0.000
Personal	3.00 (3.00-3.67)^c^	3.00 (2.67–3.33)^c^	3.00 (2.67-3.00)^a, b, d^	3.00 (2.67–3.33)^c^	0.000
Stranger Danger (1–4)*	2.00 (1.67-3.00)	2.00 (2.00-2.33)	1.83 (1.00–2.00)^d^	2.00 (1.00-2.33)^c^	0.002

Residential density scale: (1) very few houses (2) detached houses with garden, (3) attached row housing, 2-5 stories, (4) multiple apartment blocks, 6 or more stories with large spaces within buildings (5) multiple apartment blocks, 6 or more stories with very little spaces within buildings, (6) densely packed 1 story small houses, including informal settlements and slums

Other scales: higher values indicate higher land use mix (closer proximity to places), greater connection of streets, more aesthetic appearance, greater security, and less-stranger danger

Values are medians and values in parenthesis are the interquartile range of the medians (25%-75%). Differences in distribution of built environmental characteristics across neighborhood types were statistically analyzed with Kruskal‒Wallis test, pairwise comparison were done by Dunn-Bonferroni correction

Superscript numbers refer to the neighborhood type(s) with which there is a significant difference. ^a^High-SES/High-RAD, ^b^High-SES/Low-RAD, ^c^Low-SES/High-RAD, ^d^Low-SES/Low-RAD

*NEWS* Neighborhood Environment Walkability Scale, *SES* Socioeconomic status, *RAD* Residential area density

### Social cohesion

Social cohesion in neighborhoods was measured by agreement with the nine statements of ‘The Perceived Neighborhood Social Cohesion Questionnaire Brief Form’. The questions were related to trust, attachment and tolerance among people in their neighborhoods. The results are shown in Table 3. Trust among people was generally rated high, but it was lower in low-SES neighborhoods. Community attachment was generally rated high but lower in low-SES/low-RAD neighborhoods. The participants generally reported high tolerance for each other in neighborhoods, whereas tolerance was somewhat lower in low-SES/low-RAD neighborhoods. Overall, social cohesion was stronger among participants from densely populated areas than among those from low residential density neighborhoods, especially from low SES/low RAD neighborhoods.

**Table 3:** Social cohesion by neighborhood types

	High-SES/High-RAD^a^	High-SES/Low-RAD^b^	Low-SES/High-RAD^c^	Low-SES/Low-RAD^d^	*p* value
Trust					
Most people in this area can be trusted.	6.00 (4.00–7.00)^c^	6.00 (4.00–7.00)^c^	4.50 (1.00–7.00)^b, a^	5.00 (4.00–7.00)	0.020
People in this area will take advantage of you.*	3.00 (2.00–5.00)^c^	3.00 (1.00–4.00)^d^	2.50 (100-5.00)^a, d^	4.00 (2.00–5.00)^c, b^	0.003
If you were in trouble, there are a lot of people who will help you.	6.00 (5.00–7.00)	6.00 (5.00–7.00)^d^	6.00 (4.00–7.00)^d^	5.00 (4.00–7.00)^b, c^	0.036
Attachment					
Most people in this area are friendly.	6.00 (5.00–7.00)^d^	6.00 (5.00–7.00)^d^	7.00 (4.00–7.00)^d^	5.00 (4.00–7.00)^a, b, c^	0.001
People in this area have lots of community spirit.	6.00 (4.00–7.00)^d, b^	5.00 (1.00–6.00)^a, c^	6.00 (4.00–7.00)^d, b^	4.00 (3.00–6.00)^a, c^	0.000
People in this area do things to help the community.	6.00 (4.00–7.00)^d^	6.00 (5.00–7.00)^d^	6.00 (4.00–7.00)^d^	5.00 (3.00-5.50)^a, b, c^	0.000
Tolerance					
People in this area treat each other with respect.	6.00 (5.00–7.00)	6.00 (5.00–7.00)^d^	6.00 (4.00–7.00)^d^	5.00 (4.00–7.00)^c, b^	0.018
People in this area are tolerant of others who are not like them.	6.00 (5.00–6.00)^b^	6.00 (5.00–7.00)^d, a^	6.00 (4.00–7.00)	5.00 (4.00–7.00)^b^	0.002
In this area there are people who belong and some who don’t.*	2.00 (1.00–4.00)^d^	2.00 (1.00–3.00)^d^	3.00 (1.00–4.00)^d^	3.00 (2.00–4.00)^b, c, a^	0.000
Total score	5.00 (4.44–5.55)^d^	4.88 (4.44–5.56)	5.00 (4.00-5.66) ^d^	4.55 (4.11–5.11)^a, c^	0.001

Values are medians and values in parenthesis are the interquartile range of the medians (25%-75%). Differences in distribution of variables across neighborhood types were statistically analyzed with Kruskal‒Wallis test, pairwise comparison were done by Dunn-Bonferroni test

Superscript numbers refer to the neighborhood type(s) with which there is a significant difference. ^a^High-SES/High-RAD, ^b^High-SES/Low-RAD, ^c^Low-SES/High-RAD, ^d^Low-SES/Low-RAD. SES socioeconomic status, RAD residential area density

Social cohesion scale (1-7). Higher values indicate stronger agreement with the statements. *Reverse coded

### Physical activity and sedentary time

Work, transport, and leisure time-related moderate and vigorous physical activity (MVPA) was measured by the modified WHO Global Physical Activity Questionnaire, which includes an activity table with examples of activities of different ranges and intensities. The results are presented in Table 4. The most prominent part of physical activity was related to work, and the median values ranged between 25.4 and 72.4 min/day among the participants. Daily work-related MVPA was highest among participants from high-SES/high-RAD neighborhoods. Transport-related MVPA was not significant, being highest among participants from high-SES/high-RAD neighborhoods. Leisure time (recreational) MVPA was generally very low among participants (median 0.00 min/day) but lower in low-SES neighborhoods. The median values of total MVPA ranged from 40.0 to 128.9 min/day and showed a significant difference in distribution among neighborhoods; specifically, participants from high-SES/high-RAD neighborhoods had on average, 2 times higher levels of physical activity than others did. Sedentary time was considered the average daily time spent sitting and sleeping; this did not differ significantly among participants across neighborhoods. The participants spent an average of 5 h per day sitting and 8 h sleeping.

### Diet

The frequency of intake (times/week) of some common food items from the food frequency questionnaires is reported in Table 4. Consumption frequency showed significant variations in distributions across neighborhoods. Fruit and vegetable consumption was generally low, at approximately 2–3 days per week among participants. Fruit consumption was lowest in high-SES/low-RAD neighborhoods, and vegetable consumption was lowest in high-SES/high-RAD neighborhoods. Fish consumption was high in all neighborhoods, with an average of 5 days per week, and did not differ significantly among participants. Street food intake was reported an average of 3 days per week. The participants from high-SES/low-RAD neighborhoods had lower intakes of fast food, street food, sweets, sugary drinks and alcohol.

**Table 4:** Physical activity, sedentary time and diet by neighborhood type

	High-SES/High-RAD^a^	High-SES/Low-RAD^b^	Low-SES/High-RAD^c^	Low-SES/Low-RAD^d^	*p* value
Physical activity (min/day)					
Work related MVPA	72.4 (10.0 -235)^b, c, d^	29.6 (0.00–99.28)^a^	26.42 (0.00-118)^a^	10.35 (0.00-96.07)^a^	0.000
Transport related MVPA	8.35 (0.00–39.0)^d^	2.85 (0.00-42.85)	2.00 (0.00-36.78)	0.00 (0.00-10.35)^a^	0.001
Leisure time MVPA	0.00 (0.00-8.51)^c, d^	0.00 (0.00-8.57)^c, d^	0.00 (0.00–0.00)^a, b^	0.00 (0.00–0.00)^a, b^	0.000
Total MVPA	128.9 (27.1–351)^b, c, d^	64.64 (0.00-161)^a^	63.21 (0.00-188)^a^	40.00 (0.00-132)^a^	0.000
Sedentary time (min/day)					
Sitting time	300 (120–360)	300 (60–360)	300 (180–415)	300 (120–420)	0.222
Sleeping time	480 (360–480)	480 (360–480)	450 (360–480)	480 (360–480)	0.403
Diet (times/week)					
Fruit consumption	2.00 (1.00–5.00)^b^	1.00 (1.00–3.00)^a, c, d^	2.00 (1.00–5.00)^b^	2.50 (1.00–5.00)^b^	0.022
Vegetable consumption	3.00 (1.00–5.00)^b, c, d^	3.00 (2.00–7.00)^a^	4.00 (2.00–7.00)^a^	4.00 (2.00–7.00)^a^	0.001
Fish consumption	5.00 (3.00–6.00)	4.00 (3.00–7.00)	5.00 (4.00–7.00)	5.0 (3.00–7.00)	0.148
Fast food consumption	1.00 (1.00–3.00)^b^	1.00 (1.00–2.00)^a, c, d^	1.00 (1.00–3.00)^b^	2.00 (1.00–4.00)^b^	0.001
Street food consumption	3.00 (1.00–5.00)^b^	1.00 (1.00–4.00)^a ,c, d^	3.00 (1.00–5.00)^b^	3.00 (1.00–4.00)^b^	0.001
Sweet consumption	1.00 (1.00–3.00)^b^	1.00 (1.00–1.00)^a, c^	1.00 (1.00–3.00)^b, d^	1.00 (1.00–2.00)^c^	0.000
Sugary drink consumption	3.00 (1.00–5.00)^b, c, d^	1.00 (1.00–3.00)^a^	1.00 (1.00–3.00)^a, d^	1.00 (1.00–4.00)^a, c^	0.000
Alcohol consumption	1.00 (1.00–3.00)^b, c^	1.00 (1.00–1.00)^a, d^	1.00 (1.00–1.00)^a^	1.00 (1.00–2.00)^b^	0.001

Values are medians and values in parenthesis are the interquartile range of the medians (25%-75%).  Differences in distribution of physical activity and diets variables across neighborhood types were statistically analyzed with Kruskal‒Wallis test reporting the median percentiles, pairwise comparisons were done with Dunn-Bonferroni test

Superscript numbers refer to the neighborhood type(s) with which there is a significant difference. ^a^High-SES/High-RAD, ^b^High-SES/Low-RAD, ^c^Low-SES/High-RAD, ^d^Low-SES/Low-RAD

*SES *Socioeconomic status, *RAD* Residential area density, *MVPA* Moderate and vigorous physical activity

### Nutritional status

The prevalence of being overweight among the participants was much higher than the prevalence of being underweight. (Table 5). Among the men, 35.4% were overweight (BMI > 25.0), of whom 8.1% were obese, whereas only 5.9% were underweight. Among men, there were no significant differences between neighborhoods. The prevalence of overweight and obesity was almost twice as high in women as in men; 67.6% of women were overweight, 35.6% of whom were obese. The prevalence of overweight and obesity was significantly higher among women from high-SES/high-RAD neighborhoods. Among females, only 2.4% were thin.

**Table 5:** Nutritional status among males and females by neighborhood types

Nutritional status	Total	High-SES/ High-RAD	High-SES/ Low-RAD	Low-SES/ High-RAD	Low-SES/ Low-RAD	*P* value
Males						
Underweight	14 (5.9%)	2 (3.1%)	6 (9.7%)	2 (3.8%)	4 (6.8%)	0.098
Normal	139 (58.6%)	37 (57.8%)	40 (64.5%)	37 (71.2%)	25 (42.4%)	
Overweight	65 (27.4%)	19 (29.7%)	13 (21.0%)	10 (19.2%)	23 (39.0%)	
Obese	19 (8.1%)	6 (9.4%)	3 (4.8%)	3 (5.8%)	7 (11.8%)	
Females						
Underweight	9 (2.4%)	2 (2.4%)	4 (4.5%)	3 (2.7%)	0 (0.0%)	0.042
Normal	112 (29.9%)	14 (16.7%)	26 (29.2%)	35 (31.3%)	37 (41.6%)	
Overweight	120 (32.1%)	30 (35.7%)	27 (30.3%)	39 (34.8%)	24 (27.0%)	
Obese	133 (35.6%)	38 (45.2%)	32 (36.0%)	35 (31.2%)	28 (31.4%)	

The values are the numbers and percentages. Differences between neighborhood types were analyzed by Fisher’s exact test

*SES* Socioeconomic status, *RAD* Residential area density

## Discussion

The present study describes the characteristics of the built environment and the physical activity, diet and nutritional status of the residents of different SES and RAD neighborhoods in Accra, Ghana. The built environment characteristics showed significant differences in all subscales of the NEWS across the different neighborhood types. Overall, the neighborhoods were characterized by a high land use mix, which was indicated by proximity to destinations, and good connectivity of streets, especially in high-RAD neighborhoods. The aesthetics of the neighborhood and the presence and quality of the pedestrian and cycling facilities were rated low. Overall, the respondents perceived low levels of safety from traffic and crime, particularly in low-SES/high-RAD neighborhoods. Work-related, transport-related, leisure time and total physical activity levels significantly differed across neighborhood types. The most prominent part of the physical activity was work-related, and leisure-time PA was almost negligible. Total physical activity was approximately two times higher in high-SES/high-RAD neighborhoods because of greater work-related PA.

The prevalence of overweight and obesity among the study participants was much higher than that of thinness. This is consistent with previous reports, such as a GDHS 2014 survey and a systematic review of observational studies in Ghana published between 2013 and 2023, primarily among women in southern Ghana and urban settings [41, 42]. Nine of the reviewed studies were conducted in Greater Accra, but most of them concern specific population groups (university students, elderly individuals or workers), with the exception of one study which one conducted among poor urban neighborhoods. This study reported a lower prevalence of overweight and obesity than our result, but these data can only be compared with caution due to the use of different sampling methods [24].

We observed the highest prevalence of overweight and obesity among women in high-SES/high-RAD neighborhoods. Previous reports also confirmed higher rates of obesity among females and high-class residents in Accra [43–45]. Culturally, a large body size is often seen positively and is perceived as a sign of beauty, good health and happiness in marriage among African communities [46–48]. A cross-sectional study conducted in Kumasi reported that a high-calorie diet, low physical activity, and a preference for a larger body size were significant predictors of obesity in urban Ghanaian women [49].

This study revealed high street food consumption across neighborhood types, except for high SES/low RAD. This is in accordance with a study conducted in poor urban communities in Accra, Ghana, where street foods were predominant in poor neighborhoods [24]. We observed low intake of fruits and vegetables among the study participants, which could be explained by the high cost and scarce variety of food resources where fruits and vegetables are available [24]. Fish consumption was relatively high across all neighborhood types. This could be due to the geographical location of Accra, Ghana, in the coastal area where fish availability is high. Fish are the preferred source of animal protein in Ghana and play a key role in Ghanaian culinary traditions, accounting for approximately 60% of the animal protein intake within the Ghanaian diet [50, 51]. In our study, sugary drink consumption was greater in high-SES/high-RAD neighborhoods than in socioeconomically disadvantaged neighborhoods in South Africa, where a high proportion of individuals living in low socioeconomic communities purchased sugary drinks daily or weekly [52].

The built environment features were more favorable in terms of the pedestrian and bicycle environment, traffic, crime, and personal safety for neighborhood types with high socioeconomic status. A similar pattern of more favorable environments in high-income neighborhoods has been observed in studies of Western societies in America and Europe [53, 54]. Studies have revealed that both the built environment and the social environment can influence the physical activity of residents [55]. The perceived safety or prevalence of violent crime can limit outdoor physical activity and increase stress, as well as neighborhood social cohesion or collective efficacy, which can impact norms regarding diet and physical activity behaviors.

A strength of this study is the inclusion of neighborhoods with different socioeconomic statuses and residential densities, which were randomly selected. The neighborhood slum index calculated from 2010 census data for Ghana was the neighborhood SES indicator. We were able to measure the perceived built neighborhood characteristics with the NEWS questionnaire validated for the African environment. The assessment of nutritional status was based on measured weight and height data, which are less biased than self-reports are. While data on physical activity and diet were self-reported, they can be under- or over-reported, which is a limitation of this study. Physical activity was measured using the modified WHO GPAQ [40], which is an extension of the original GPAQ with an activity table showing activities of different ranges and intensities, which helped in a more accurate estimation of physical activity. Our study simultaneously measured the built environmental and social characteristics of neighborhoods with various aspects of healthy lifestyle indicators, including physical activity, diet and nutritional status, as well as participants’ sociodemographic characteristics. This allows us to conduct more analytic studies to explore the associations between the built environment and individual characteristics.

## Conclusions

In conclusion, there were significant differences in built environment characteristics, physical activity, diet, and nutritional status across neighborhood types. The findings highlight the need for multifaceted interventions to promote health equity and improve population health outcomes across diverse neighborhood types. There should also be a need for policies at the local, national, and international levels that prioritize health equity and address the social determinants of health, including access to safe and healthy built environments, equitable access to recreational amenities, and the promotion of healthy lifestyle behavior. Urban planning interventions, such as enhancing sidewalks, bicycle facilities, streetlights, and street crossings to promote safer walking and cycling, need to be implemented to improve the characteristics of the built environment in low-income neighborhoods. There is a need to develop tailored interventions to promote physical activity, particularly in low-density areas, through initiatives that enhance street connectivity and provide accessible opportunities for exercise and recreation. Further analysis is needed to ascertain the magnitude of the associations among built environment characteristics, physical activity, diet, and nutritional status by neighborhood types. These insights can provide valuable information for governments, policymakers, urban planners, and public health officials to develop targeted interventions that address specific challenges in different types of neighborhoods, ultimately promoting healthier living environments in Accra, Ghana.

## Data Availability

The datasets that support the findings are available from the corresponding author upon request.

## Abbreviations

BMI: Body Mass Index
GDHS: Ghana Demographic Health Survey
GIS: Geographic Information System
GPAQ: Global Physical Activity Questionnaire
GPS: Global Positioning System
IQR: Inter Quartile Range
MVPA: Moderate to Vigorous Physical Activity
NEWS: Neighborhood Environment Walkability Scale
P-NSC-BF: Perceived Neighborhood Social Cohesion Questionnaire Brief Form
QGIS: Quantum Geographic Information System
RAD: Residential Area Density
SES: Socio Economic Status
SPSS: Statistical Package for the Social Sciences
WHO: World Health Organization
